# A Unifying Perspective on Perception and Cognition Through Linguistic Representations of Emotion

**DOI:** 10.3389/fpsyg.2022.768170

**Published:** 2022-05-31

**Authors:** Prakash Mondal

**Affiliations:** Department of Liberal Arts, Indian Institute of Technology (IIT), Hyderabad, India

**Keywords:** perception, cognition, emotion, linguistic representations, cognitive systems, autonomous systems

## Abstract

This article will provide a unifying perspective on perception and cognition *via* the route of linguistic representations of emotion. Linguistic representations of emotions provide a fertile ground for explorations into the nature and form of integration of perception and cognition because emotion has facets of both perceptual and cognitive processes. In particular, this article shows that certain types of linguistic representations of emotion allow for the integration of perception and cognition through a series of steps and operations in cognitive systems, whereas certain other linguistic representations of emotion are not so representationally structured as to permit the unity of perception and cognition. It turns out that the types of linguistic representations of emotion that readily permit the desired unity of perception and cognition are exactly those that are linguistically encoded emotive representations of everyday objects, events, and things around us. It is these ordinary objects, events and things that provide the scaffolding for task-dependent or goal-oriented activities of cognitive systems including autonomous systems. In this way, cognitive systems can be saliently tuned to the outer world by being motivated and also subtly governed by emotion-driven representations. This helps not only tie together perceptual and cognitive processes *via* the interface between language and emotive representations, but also reveal the limits of emotive representations in amalgamating perceptual and cognitive processes in cognitive systems.

## Introduction

This paper aims to offer a conceptual analysis of parallels and formal similarities between linguistic representations of emotion and perception, thereby offering a way of integrating perceptual representations and cognitive representations (especially linguistically encoded representations of emotion) for cognitive systems.^1^ At this point, it is essential to clarify the terminology to be used. By integrating the notions of representation and concepts in Connell and Lynott (2014), *cognitive representations* can be demarcated as specific, situated, contextually driven, or context-free structures and/or schemas that are realized as conglomerations of neural activation patterns for the interpretation and evaluation of objects, events, situations in the outer world, and include both online (part of current experience) and offline (not part of current experience) information. Cognitive representations in this sense include experiences or conceptual representations that can be re-activated/triggered in an offline format (for example, experiences of rain that can be re-activated when there is no rain) and hence may draw upon neural resources across perceptual, motor, emotive/affective, linguistic, and other areas. Crucially, cognitive representations have been traditionally thought to be amodal, proposition-type representations in the mind (Fodor and Pylyshyn, 1988) and are supposed to exclude sensory-motor and emotive/affective representations, although this is what is challenged in embodied theories of cognition (see Barsalou, 2008; Shapiro, 2019). *Linguistic representations* and *perceptual representations* refer, respectively, to cognitive representations coded in language and representations grounded in the sensory systems. When emotive/affective representations are expressed in natural language and assume the typical character of cognitive representations, we may call such representations *linguistically encoded representations of emotion*. Now we turn to the main issue.

Despite representational and domain-specific differences between emotion and perceptual systems, there are some formal correspondences at the representational level between emotion and perception. Given that emotions exhibit facets of both non-cognitive processes (Damasio, 1994, 2003; Oatley and Johnson-Laird, 2002; Zinck and Newen, 2008; Panksepp and Biven, 2012) and cognitive mechanisms (Scherer, 1984; Ortony et al., 1988; Lazarus, 1991), any kind of emotion-perception homologies can help uncover the properties and aspects of convergences between cognition and perception at a fundamental level. On the one hand, much of mainstream cognitive science often segregates perception from the rest of cognition (see Fodor, 1998; Pylyshyn, 1999; Burge, 2010; Block, 2014, 2018; Firestone and Scholl, 2016). On the other hand, recent research reveals significant convergences and overlaps between perception and cognition (Tacca, 2011; Clark, 2013, 2016; Montemayor and Haladjian, 2017). Linguistic encoding of emotive representations will play a special role in showing patterns of correspondences between perception and cognition since linguistically encoded representations of emotion are cognitive representations (Mondal, 2016a) and these representations also evince a perceptual character in view of emotion-perception homologies. Invariances across the perception-cognition spectrum can help us understand the dimensions of processing and representation in cognitive systems that deal with the computational load of attending to only relevant detail from within a morass of irrelevant detail in the perceptual environment. This paper will show that a specific class of linguistically encoded representations of emotion readily admit of the desired unity of perception and cognition, and if so, it is the formalization of such representations that *may* hold the key to task-directed perception in AI systems, crucially because these representations will carry aspects of *feature salience* as part of the linguistic (and hence cognitive) encoding of emotive contents. These cognitive representations of emotive contents understood as linguistically encoded representations of emotion, are special because they can *selectively* direct cognitive systems including AI systems to certain unique and relevant aspects of the outer world. Cognitive representations of emotive contents are often valenced,^2^ infused with affective valuation, and also amenable to inferential calculations. In virtue of being saliently tuned to those specific linguistically shaped emotive/affective representations of objects, things, and events that are distilled from perceptual representations, cognitive systems can discriminate between what is perceptually significant and what is or may not (see Pessoa, 2019, p. 164–165). Thus, the problem of how to reduce the perceptual complexity of the outer world for discrimination, classification, and task-switching can be partly tackled by building cognitive representations of emotive contents that allow for manipulations for inferences, reasoning, and planning. The aim of this paper was to show what such representations are, and in doing so, attempts to offer directions for formulating coordination principles between perception and cognitive representations *via* the route of emotive representations.

This paper is structured as follows. “Two Types of Linguistic Representation of Emotions” will discuss the distinction between two types of linguistic encoding of emotions and their links to cognitive representations and processes. “Emotion-Perception Parallels” will focus on the parallels between emotion and perception in order to flesh out the link between perception and cognition *via* the route of emotions that *partially* partake of aspects of cognition. Then “The Formalization of the Linguistic Encoding of Emotive Contents of Extensional Equivalence and Perception-Cognition Coordination” will show how to formalize the type of linguistic encoding of emotions that readily admits of the desired unity of perception and cognition, with a view to highlighting its implications for coordination principles between perception and cognitive representations and/or processes in cognitive systems. Finally, “Concluding Remarks” will offer concluding remarks.

## Two Types of Linguistic Representation of Emotions

The representational contents of emotions are what emotions stand for or are about or simply represent. Hence representational contents of emotions are *part* of emotive/affective representations in the brain as external or internal stimuli are transformed into meaningful experiences of things, events, situations. There is neuroscientific evidence that such contents are encoded in medial prefrontal cortex and/or insula and the temporal–parietal memory circuits (see LeDoux, 2020: R620-621). The frontal lobe has also been found to be involved in this (Freeman, 2000). Work from the philosophy of language and mind to be drawn upon below aligns well with this. The emotive contents characterize the *intentional* contents^3^ of emotions. The intentional contents of emotions can include both *thin contents* characterized by the relational structure inherent in the representational contents of emotions, and also *thick contents* that include sensory qualities of experiences, subjective qualities of experiences, particular affect or feelings associated with experiences, and crucially modes or ways of viewing or experiencing (also called Fregean contents) (Georgalis, 2006; Montague, 2009). Both these types of emotive contents are encoded in linguistic expressions/representations of emotions. They can also be distinctly segregated in linguistic expressions of emotions. Some representative cases can exemplify this.

1. Roy is jubilant at his victory over Joy.
2. Maya is not happy about the purchase of the truck.
3. They have been quite excited about the arrival of the machinery from Russia.
4. Ron is fond of Superman.
5. Danny loves the City of Light.
6. We hate the guest of honor in today’s event.

First of all, in (1) Roy is jubilant at his victory over Joy but not at Joy’s defeat even if both Roy’s victory and Joy’s defeat are *logically* equivalent and so intersubstitutable. The special nature of emotive contents in this case is such that even if Roy is made to understand that his victory over Joy and Joy’s defeat are one and the same thing, the relevant inference cannot be so executed in Roy whose jubilation is oriented *only* toward his victory over Joy but not toward Joy’s defeat *per se*. Similarly, in (2) if Maya is not happy about the purchase of the truck, it does not follow that Maya is not happy about the sale of the truck from whatever place it was sold. Likewise, in example (3) they have been excited about the arrival of the machinery from Russia but not about the departure/dispatch of the machinery from Russia. In all these three cases, the special nature of emotive contents expressed in language makes it hard and well-nigh impossible to get inferences and reasoning to affect and intersperse with emotions. On the basis of such observations, Montague (2009) has notably contended that the intentionality of emotion is unique and cannot be reduced to that of other mental states, such as beliefs, reasoning, or cognitions. That is because the intentional content of emotions is intimately associated with the experiential valuation and modes of viewing or experiencing things. It is to this experiential valuation and mode of experiencing that we appeal when referring to thick contents. Thin contents, on the other hand, can be easily characterized, by means of a *relation* on the set of things that participate in any emotive attitude. Thus, for example, the schema in (7) captures the basic thin content of (1–3).

7. *s*R*t* = *s* is related by R to *t*

Here, *s* is the agent/actor who holds an emotive attitude and *t* is the thing toward which the attitude is held by *s*. The relation R is an *intentional* mode applicable to each specific instance of an emotive predicate—"jubilant,” “not happy,” and “have been excited” in (1), (2), and (3), respectively, each instantiate R. An emotive attitude is thus exemplified by an intentional mode which is a psychological event that may be perceiving, thinking, believing, or something else. Interestingly, adopting an intentional mode has been found to be supported by a mentalizing system in the brain consisting of dorsal and ventral areas of medial prefrontal cortex, posterior cingulate cortex, and temporal poles (Spunt et al., 2011).

Interestingly, Montague points out that the resistance of emotive contents to inferential processes of cognition is not exhibited by all contexts of linguistically encoded expressions of emotive contents. Some cases of linguistic encoding of emotive contents distinctively diverge from the pattern in (1–3). The examples in (4–6) can help figure out what these contexts are. Given example (4), it may now be observed that Ron is actually fond of Clark Kent if Ron eventually recognizes that Superman and Clark Kent are one and the same person. Likewise, in (5) it may turn out that Danny learns that Paris is the City of Light, and if that happens, Danny then loves Paris. Similar considerations apply to (6) as well, because if the speakers hate the guest of honor in the specific event talked about, these speakers will also hate Mr. X (who is a neighbor of the speakers, let us suppose) if Mr. X turns out to be the guest of honor in that event. In such cases, the resistance to inferences from the rest of cognition is not that strong. One proviso is in order here. When we say that the resistance to inferences in cases like (4–6) is not that strong, it may be a matter of degree. If the actor(s) concerned [in examples (4–6)] already have an opposite disposition toward Clark Kent or Paris or Mr. X, the resistance can be strong. Another example can clarify the point here. Let us suppose that someone believes that Mr. Hyde is a rogue and hence abhors him because Mr. Hyde has hit this person once. In addition, this person also knows Dr. Jekyll who lives close by. Now if the person comes to discover all of a sudden that Dr. Jekyll is none other than Mr. Hyde, this person cannot but believe that Dr. Jekyll is a rogue, no matter how difficult it may be for this person to take in this fact. This indicates that the resistance to reasoning and associated inferential processes sort of breaks down in this case. Notwithstanding this point, if the person concerned not only knows Dr. Jekyll but also likes him, then this person may not readily abhor Dr. Jekyll just because he has turned out to be none other than Mr. Hyde. It seems that some sort of indeterminacy may operate here. However, the very fact of the person’s resistance to feeling hatred for Dr. Jekyll along with the concomitant indeterminacy *presupposes* that the relevant inferences have already gone through. That is, the relevant inferences regarding the identity of Dr. Jekyll and Mr. Hyde must have gone through. Hence the resistance to reasoning and inferential processes *cannot* be very strong here.

Montague argues that this kind of inferential sensitivity is eminently present in contexts of belief and cognitions but seems to collapse for other emotional contexts, as in (1–3). Montague’s position is that the intentional content of emotion cannot be generally or uniformly reduced to that of other mental states like beliefs, cognitions. But one may wonder why this difference occurs. By analyzing these emotive contexts closely, Mondal (2016a) has contended that the fundamental difference between the two linguistic contexts of emotive encoding lies in the logical form of the noun phrases that characterize the intentional object toward which the emotive attitude is directed. That is, noun phrases “his victory over Joy,” “the purchase of the truck,” and “the arrival of the machinery from Russia” in (1–3), respectively, have a special equivalence relationship with their counterparts (that is, with “Joy’s defeat,” “the sale of the truck,” and “the departure/dispatch of the machinery from Russia”). The equivalence concerned is one of *entailment* and thus logically necessary. If X gains victory over Y, it automatically follows that Y is defeated by X; if X is bought, it follows that X must have been sold (by someone); if X arrives from Y, then it is necessary that it goes from Y to X. In contrast, the equivalence of terms between “Superman” and “Clark Kent,” as in (4), is not logical. The equivalence concerned is merely *extensional* and hence based on the physical extension of the terms involved. In other words, “Superman” and “Clark Kent” both converge on the singular *physical* extension of the person who is known by the two names. The same argument applies to the equivalence between “the City of Light” and “Paris” in (5) and also to that between “the guest of honor” and “Mr X” (as an example) in (6). Even though it may be thought that the extensional equivalence applies to specific objects, closer scrutiny may reveal that we are surrounded by all sorts of entities that can have different names/terms. One’s father and mother’s husband have the same extension; a person eating at Table 5 in a restaurant and Mr. Z (as an example) can have the same extension; one’s favorite dish and lasagna, for example, have the same extension; the deadly virus that has invaded human lives now and coronavirus have the same extension; a blue-colored book on cognition in the library and Frank George’s book “Cognition,” for example, have the same extension; the toilet paper guy in the office and Mr. Y (as an example) are one and the same person. In all these cases, the equivalence is anchored in our commonsense knowledge and hence may change over time as new roles are taken by new entities (such as the president of a country). It is this type of equivalence that readily permits emotive contents to be affected by inferences. Perception in cognitive systems can be guided by the recognition of such equivalence, for it obviates the need to pay attention to irrelevant sensory-cognitive features and properties of the entities concerned. This warrants recognizing the other label(s) for the same entity. Emotive contents in the case of extensional equivalence, in virtue of being guided by cognitions, can be tuned to predictive coding because it also helps modulate emotional contents in accordance with the internal and external causes of changes within the internal realms of cognitive systems (Seth, 2013). Given our case for emotive contents tuned to extensional equivalence, entities with different terms or labels can be causes of changes within cognitive systems that can assume recurrent patterns so that the equivalence is instantly recognized and what is perceptually significant can be isolated from the rest.

## Emotion-Perception Parallels

Emotion and perception have a number of formal and organizational similarities. For one thing, if sensory perception has certain characteristics that are shared with emotions, this *in itself* would be indicative of the common threads in emotion and perception. For another, if the bipartite division in emotive contents can be found in perception as well, this can buttress the case for emotion-perception parallels from the perspective of emotions. Thus, homologous cognitive structures at formal and functional levels of emotion and perception can exist. In fact, *constructivist* theories of emotion have amassed a wealth of empirical evidence showing that states of perception cannot be told apart from those of emotion because language shapes sensory experiences into experiences of emotion and also vice versa, especially in emotion perception (Lindquist et al., 2015). Beyond mere analogies between emotion and perception, there is also neuroscientific evidence that the functional connectivity of the cortical and sub-cortical circuits responsible for both sensory-perceptual processes and emotion is characterized by overlapping networks (Pessoa, 2017) and involves the same pathways at the level of general networks of cognition (LeDoux and Brown, 2017; LeDoux, 2020).

Interestingly, Searle (1983) has argued that the formal structure of intentionality (when applied to emotions in our case) and visual perception is common, in that both in essence are states that have the property of being directed to something. Solomon (1978) and Maiese (2011) have also provided support for the view that emotions and perceptions are alike in nature and form. From another perspective, Roberts (1995) has maintained that emotions are like perceptions. Just as perception can be veridical or non-veridical in terms of aspects or dimensions, such as situation, importance, type, and object, emotions can have a similar character. For instance, one can be thrilled about the falling star by associating it with wish fulfillment even if it is a false indication (an error in terms of situation misrepresentation). Likewise, one can be angry with someone for a trivial offence (an error in terms of importance misrepresentation). Equally possible is a case in which one is nervous about a thing but in fact excited about it (an error as a consequence of type misrepresentation). Finally, a person can be frightened of a game but is actually fearful of himself/herself (an error due to object misrepresentation). At this juncture, it is also worthwhile to recognize that emotion and perception can be dissimilar too, for emotions have cognitive ingredients or elements and may have a normative character due to cultural and subjective influences, they resemble one another in terms of their immediacy, intentional property, *quasi*-modularity and impoverished sensitivity to inferences in many cases (see Salmela, 2011). In any case, the underlying affinity between emotion and perception cannot be sidelined, because emotions share the characteristics of interoceptive states displayed by perceptions (Prinz, 2006), in that emotions are interoceptive states characteristic of perceptual systems in virtue of *registering* rather than representing bodily changes.

Just as the characteristic properties of sensory perception at basic levels of organization can be found to be reflected in emotion, the essential facets of emotion can be mirrored in perception. Emotion and perception can work in an integrated manner when emotion influences perception (such as height perception) by way of providing embodied information about actions (see Zadra and Clore, 2011). Perception thus comes to exhibit aspects or features that are inherited from or true of emotions (see for discussion, Tyng et al., 2017; Niedenthal and Wood, 2019). Against a backdrop of this understanding, we may now figure out if the bipartite division in emotive contents is also mirrored in some form in perception. From one perspective, sensory perception can be non-transitive in its formal structure, especially if we find X and Y to be perceptually indistinguishable and then Y and Z to be also perceptually indistinguishable, *and yet* we may not find X and Z indistinguishable (Van Deemter, 2010). This may hold when degrees of differences between X and Y on the one hand and between Y and Z on the other add up, thereby making up a sizable difference that is ultimately detected in perception. An example may help understand this better. Suppose that we have three toys, namely, A, B, and C that have the same size, shape, color, substance and form. Any difference between these toys is only at a minute level of detail not easily detectable through our eyes. But whatever minuscule physical difference exists between A and B is a bit smaller than that between B and C. Hence, A and B may look indistinguishable and so may B and C on independent grounds. But the difference between A and B and that between B and C amount to a recognizable difference that is perceptually recognizable. Thus, this helps recognize a difference between A and C. This may extend to other modalities of sensory perception as well (see Mondal, 2016b.). Now this kind of non-transitivity can straightforwardly apply to the emotional contexts of (1–3) in “Two Types of Linguistic Representation of Emotions”. The case of logical equivalence and the concomitant absence of inferential sensitivity are most appropriate for the exploration into the formal structure of emotions by way of extrapolations from the formal organization of perception. We may consider the case of (1) as a representative example. If Roy’s victory over Joy (say, A) and Joy’s defeat (say, C) do not look the same in the emotional context of utterance of (1), it is plausible that there is a representation of, say, B, intermediate between A and C such that Roy finds A and B on the one hand and B and C on the other equivalent in emotional valuation in the context of (1). Let us also suppose that this intermediate representation B is the final checkmate move in chess in which Roy won a victory over Joy. Now *for Roy,* his victory over Joy (A) and the final checkmate move (B) may be indistinguishable in emotional valuation and then the final checkmate move (B) and Joy’s eventual defeat (C) may also turn out to be so indistinguishable. If this holds, the transition from A through B to C can gather and accumulate substantial differences in emotional valuation between A and C. This would be translated into a kind of non-transitivity in the linguistic encoding of emotive contents. We may now turn to the case of extensional equivalence in the encoding of emotive contents in order to see if any other property of perception is also congruent with extensional equivalence with its inferential sensitivity.

**Figure 1:**
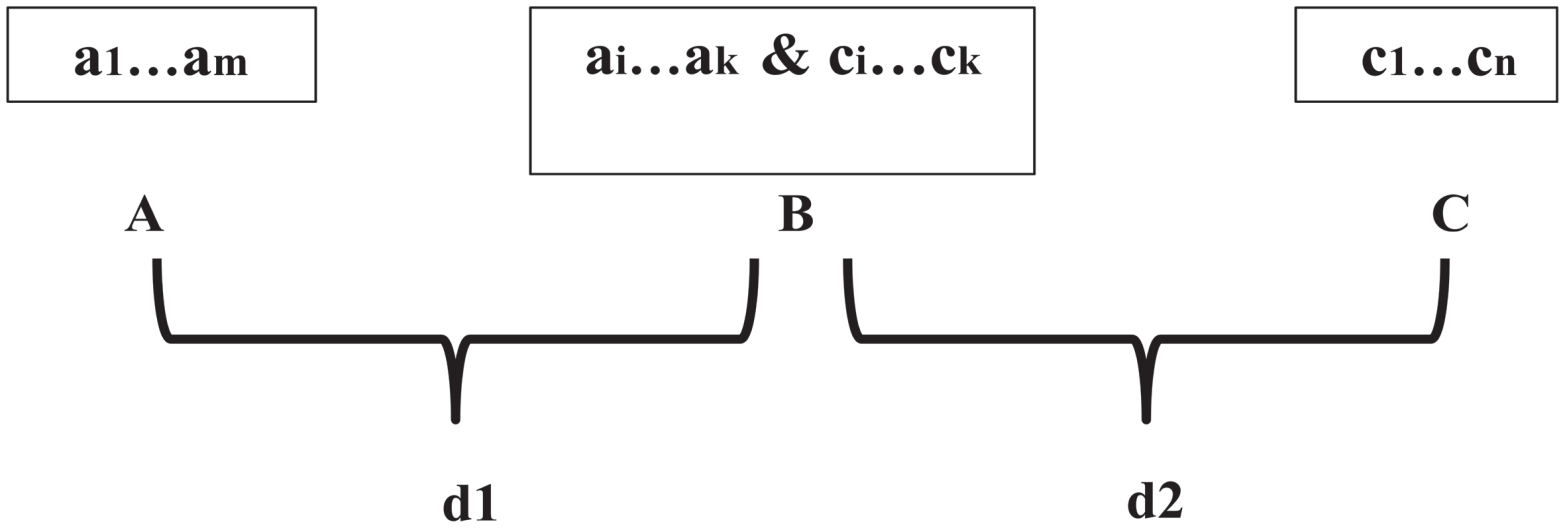
Transitivity in emotive contents in cases of (4–6).

From another perspective, sensory perception can sometimes be transitive as well when an intermediate thing or representation facilitates the recognition of the identity between two otherwise dissimilar entities in emotional valuation. In simpler terms, non-transitivity in sensory perception may presuppose transitivity in sensory perception. Transitivity in sensory perception would guarantee that if there are three things, say A, B, and C and if A is found to be similar to, or indistinguishable from, B and B is in turn found to be indistinguishable from C, then A and C would look indistinguishable from one another. For example, two cars—C1 and C2—may be found to be similar or indistinguishable and then C2 may also be found to be similar to, or indistinguishable from, a third car C3. In this case, one may find C1 and C3 to be perceptually similar or indistinguishable. This suggests that C2 must have perceptual features or properties that are shared with both C1 and C3 in order that the similarity or identity in question can be perceptually established. This can have significant consequences for the way two things are registered and/or conceptualized in emotional valuation in cases like (4–6). If we apply this line of reasoning to example (4) in “Two Types of Linguistic Representation of Emotions”, it becomes evident that the identity between “Superman” and “Clark Kent” as terms has to be inferentially established *via* an intermediate representation. The intermediate representation must have to capture the essential attributes of both Superman and Clark Kent in order for the appropriate inference to establish the desired identity. Similar considerations apply to example (5). For Danny to love Paris, he has to mentally construct an intermediate representation that encodes the essential emotionally relevant attributes of both the City of Light and Paris once he learns that Paris is the City of Light. Likewise, in example (6) for the speakers to eventually hate Mr. X, an intermediate representation encapsulating the emotionally relevant attributes of the guest of honor of the specific event and Mr. X may be built. It needs to be noted that the psychological construction of an intermediate representation is eminently necessary, especially when the salient emotionally relevant attributes of any two entities whose identity is supposed to be established are somehow incongruent in valuation. As already discussed in “Two Types of Linguistic Representation of Emotions”, it is plausible that even though the guest of honor in example (6) may be hated by the speakers, Mr. X may be revered by them. In such a situation, the *common* emotionally relevant attributes of both the guest of honor and Mr. X, if there are any, have to coalesce into some form in order for the integration of the uncommon attributes of both the guest of honor and Mr. X to occur. Besides, even if the salient emotionally relevant attributes of any two entities are not otherwise incongruent in valuation, the common emotionally relevant attributes or features have to be re-constructed anyway, precisely because the identity between those two entities is not recognized in advance. In view of these considerations, the mirroring of transitivity of sensory perception in emotions can be schematized in terms of the diagram in Figure 1.

**Figure 2:**
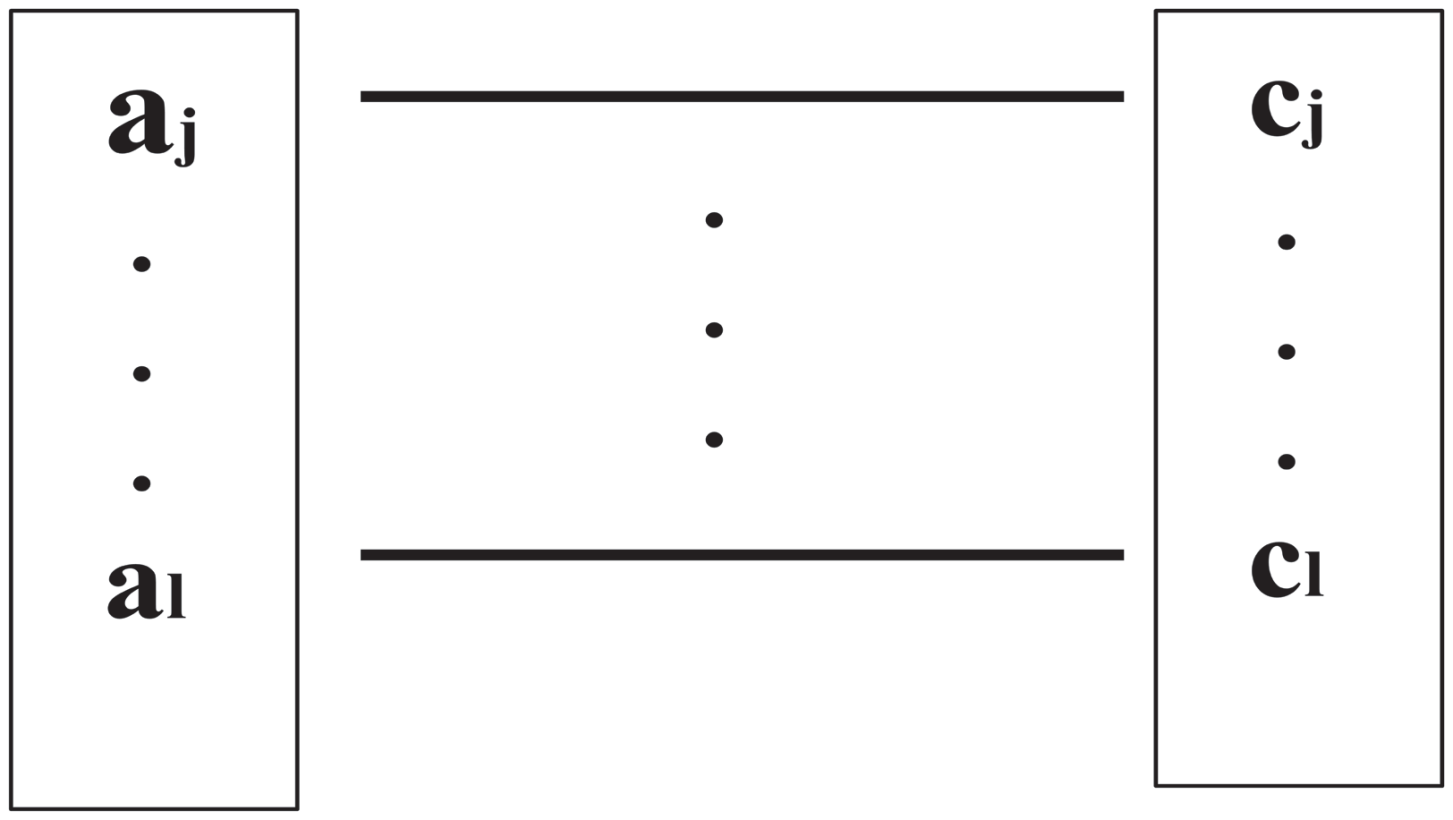
The isomorphism between (a subpart of) a_i_…a_k_ from A and (a subpart of) c_i_...c_k_ from C.

Here, a_1_…a_m_ & c_1_…c_n_ are the salient emotionally relevant attributes of A and C, respectively, and a_i_…a_k_ & c_i_…c_k_ symbolizes the integration of the most essential perceived attributes of both A and C into B, the intermediate representation. Note that *i…k* may or may not coincide with *1…m* and *1…n*, because these sequences may not coincide with each other when incompatible features/attributes from A and C, if put together or juxtaposed, are in need of elimination (this will become clearer in the next section). Also, d1 and d2 in Figure 1 are the *degrees* of similarity or *indistinguishability* that make A and B on the one hand and B and C on the other indistinguishable, respectively, for the pairs (A, B) and (B, C). This indicates that there are uncommon attributes in A and C that make A and C look distinguishable, but they may need to be unified in B too. Significantly, the integration of all common attributes of both A and C, if any, into B has to be achieved *via* the establishment of some sort of *isomorphism* between a subpart of a_i_…a_k_ (say, a_j_…a_l_) from A and a subpart of c_i_…c_k_ (say, c_j_…c_l_) from C since not all of a_i_…a_k_ or c_i_…c_k_ may be common between A and C. This is tantamount to the recognition of the commonalities between A and C. This can be shown in Figure 2.

The common attributes of A and C in B may also be conceived of in terms of a feature structure [f^b^_1_…f^b^_k_] such that this feature structure of B can *subsume* the feature structure of either A (say, [f^a^_1_…f^a^_m_]) or C (say, [f^c^_1_…f^c^_n_]), given that the *subsumption* relation (symbolized by “⊑”) is used to describe a feature structure containing a subset of the information contained in another feature structure (Shieber, 2003). Thus, [f^b^_1_…f^b^_k_] ⊑ [f^a^_1_…f^a^_m_] and [f^b^_1_…f^b^_k_] ⊑ [f^c^_1_…f^c^_n_] hold true. Therefore, unless incompatible feature structures from A and C are involved, we have that B = [f^a^_1_…f^a^_m_] ⊔ [f^c^_1_…f^c^_n_], where “⊔” symbolizes *unification* given that [f^a^_1_…f^a^_m_] ⊑ B and [f^c^_1_…f^c^_n_] ⊑ B. This formulation is going to be very helpful for the formalization of the linguistic encoding of emotive contents in the case of extensional equivalence. This is what we shall turn to now.

## The Formalization of the Linguistic Encoding of Emotive Contents of Extensional Equivalence and Perception-Cognition Coordination

In order to make explorations into the coordination principles between perception and cognitive representations and processes in cognitive systems including autonomous systems, we need to formalize the linguistically encoded emotive representations of extensional equivalence because it readily applies to everyday objects, events, and things around us. This would also offer significant insights into the nature and form of integration of perception and cognition. With this in mind, we may now move on to describe emotive representations of extensional equivalence. Cases like (4–6) require warrant at least a basic distinction between the feature structures of two objects that are described in a certain way in the linguistic encoding of emotions. For our purpose, we may adopt the labels used for transitivity in emotive contents in “Emotion-Perception Parallels”. So if A and C are the two terms of description of these two objects whose identity is in question, they may be specified with respect to a relevant emotive attitude. Let this attitude be denoted by R^E^, which is mathematically a relation. Thus, an agent (or subject) S would first hold what (8) specifies.

8. _S_R^E^_A_

It is easy to note the parallel between (8) and (7) as the relation R^E^ in (8) relates an agent S to its intentional object. Then the same agent is supposed to hold what (9) specifies *via* the execution of the appropriate inference.

9. _S_R^E^_C_

That is, the appropriate inference should trigger a transition (designated by “→”) from _S_R^E^_A_ to _S_R^E^_C_ when S finds out that A = C, as is shown in (10).

10. _S_R^E^_A_ → _S_R^E^_C_ when _S_R^E^_A_ = _S_R^E^_C_

Now it is essential to specify how A and C *initially* look distinct infused with distinguishable emotional valuation features. If we make reference to example (4), the emotional valuation features of A and C (that is, [f^a^_1_…f^a^_m_] and [f^c^_1_…f^c^_n_]) must be initially distinct *for* Ron. If A = Superman and C=Clark Kent, the distinguishable valuation features of A and C can be coded in terms of *qualia* properties in Generative Lexicon Theory (Pustejovsky, 1995). Qualia properties are constituted by *formal* (the basic ontological category of entities), *constitutive* (the relation between an entity and its constituent parts), *telic* (the purpose or function of an entity), and *agentive* properties/features (an entity’s coming into being). With the help of qualia structures, we can now distinguish “Superman” from “Clark Kent” for our S = Ron, as shown in (11).

11. Superman (A): *non-human* • *good-superpowerful-being* ⨂ *_Telic_ save_humans*

Clark Kent (C): *human* ⨂ *_Telic_ report* ⨂ *_Agentive_ nil.*

The tensor operator ⨂ signals the composite form of the qualia properties of the “Superman” and “Clark Kent” in (11), and *non-human* • *good-superpowerful-being* represents a complex qualia type as a dot product (see Pustejovsky and Jezek, 2008). Importantly, when _S_R^E^_A_ holds, we may suppose that the emotive attitude toward A is toward the qualia structure of “Superman” as represented in (11), and also that when _S_R^E^_C_ holds the emotive attitude toward C is actually toward the qualia structure of “Clark Kent” in (11). The role of inference is to link *unify* the qualia structures of A and C *via* B. When Ron discovers that A is actually C, the incompatible qualia structures of A and C have to coalesce into B in some way. While *_Telic_ save_humans* and *_Telic_ report* are compatible because they can be unified into a single structure, *non-human* • *good-superpowerful-being* and *human* are incompatible. Therefore, upon discovering that A is actually C, Ron’s construction of B will look like (12), which also shows the accommodation of compatible qualia properties and the elimination of incompatible qualia properties.

12. B: *non-human* • *good-superpowerful-being* ⨂ *_Telic1_ save_humans* ⨂ *_Telic2_ report*

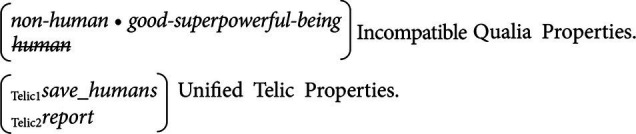


The distinguishable valuation features of “the City of Light” and “Paris” in (5) can also be characterized in terms of qualia structures, as shown in (13).

13. The City of Light (A): *space • beautiful-lights* ⨂ *_Constitutive_ {buildings, people, roads…}* ⨂ *_Telic_ live_in*

Paris (C): *space • capital-of-France* ⨂ *_Constitutive_ {buildings, people, roads…}* ⨂ *_Telic_ live_in.*

The incompatibility between the qualia structures of A and C for example (5) lies in the formal qualia properties of A and C which, we suppose, hold for Danny (=S). If so, the form of B for Danny can be specified in (14).

14. B: *space • beautiful-lights* & *space • capital-of-France* ⨂ *_Constitutive_ {buildings, people, roads…}* ⨂ *_Telic_ live_in*

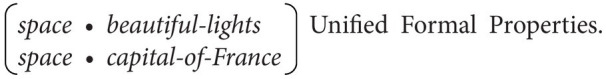


Properties, such as *good-superpowerful-being* (for (4)), *beautiful-lights* (for (5)), are called *conventionalized attributes* that capture the features and/or attributes of entities as they are experienced (Pustejovsky and Jezek, 2016). Likewise, the distinguishable valuation features of “the guest of honor” and “Mr X” in (6) would be what (15) schematizes.

15. The guest of honor (A): *human • object-of-despise* ⨂ *_Telic_ address-in-event* ⨂ *_Agentive_ invite*

Mr. X (B): *human • neighbor* ⨂ *_Agentive_ nil.*

If we suppose for the speakers of the utterance in (6) the valuation features of “the guest of honor” and “Mr X” are what (15) specifies, the form B assumes can be shown in (16).

16. B: *human • object-of-despise* & *human • neighbor* ⨂ *_Telic_ address-in-event* ⨂ *_Agentive_ invite*

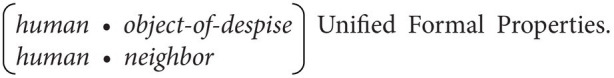


Interestingly, for an (autonomous) agent the meaning of B in (12)/(14)/(16) is nothing other than the *implication* (*Impl*) relevant to a particular set of goals G formed at time *t + x* of a particular agent S with particular knowledge K in the *i-*th circumstance C_i_ which is a state of the world at some point of time after *t* (Thórisson et al., 2016, p. 111). This is formulated in (17). An implication on this view is a number of computed deductions D. The set of all implications in a circumstance constitutes the meaning of a datum *d_t_* that can be an event or perception of something.

17. *Impl*(d_t_, S(G)_t + x_) = D(d_t_, C_i_, (K_S_, G_S_, C_S_)_t + y_)

**Figure 3:**
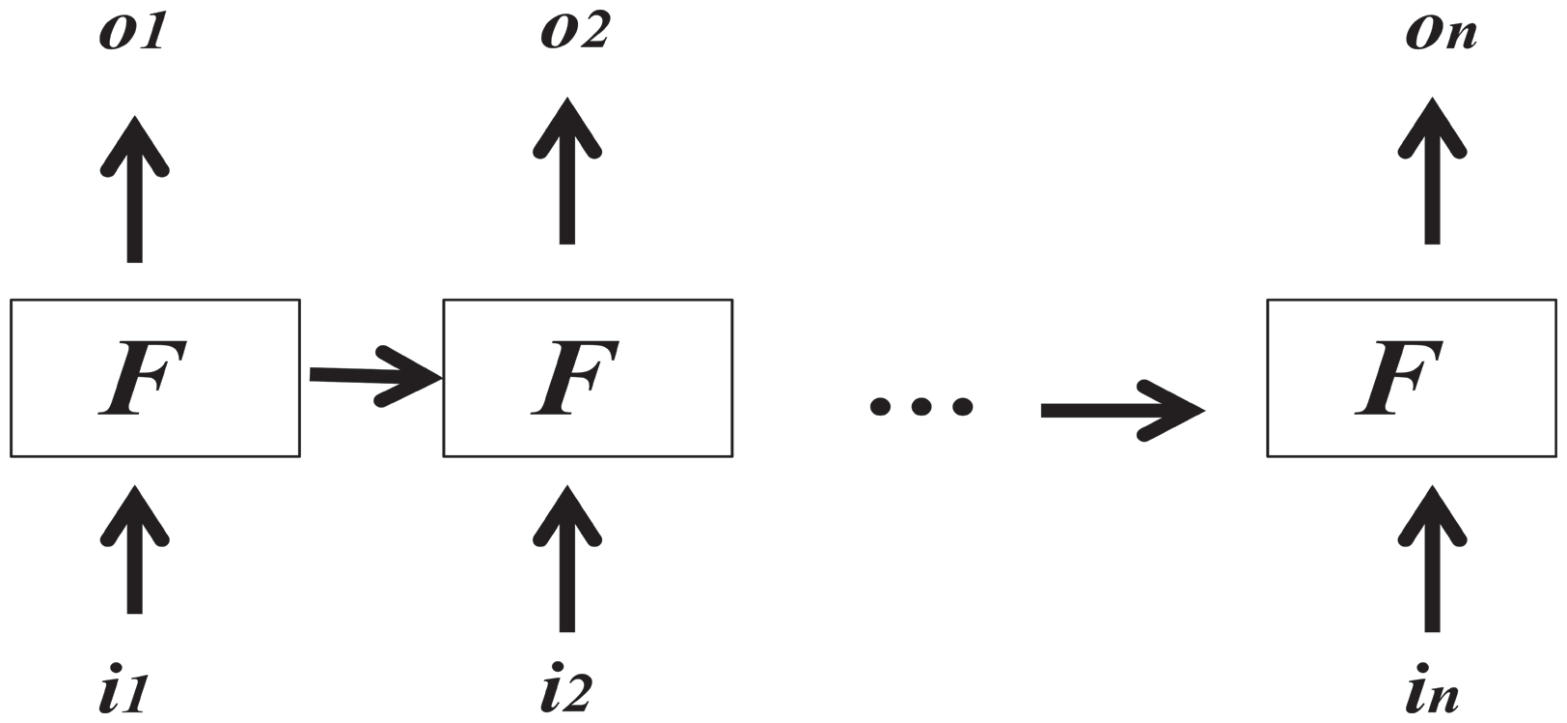
A recurrent neural network in backpropagation modeling _S_R^E^_A_ → _S_R^E^_C_.

Here, *t + x* and *t + y* may refer to different points in time after *t* because the implications may be relevant for S at future points of time too. Most importantly, *d_t_* in the context of the current work would be the discovery that A is C, and then the unification of qualia properties and/or the elimination of incompatible qualia properties in B are *nothing but* representations of D. That is, the specifications of the common and incompatible qualia properties in B by way of unification and/or elimination are deductions that help arrive at the conclusion that A = C. Such deductions in the form of inferences are constitutive of (minimal) understanding for an (autonomous) agent/cognitive system (see Thórisson and Helgasson, 2012; Mondal, 2021). Equipped with this understanding, we think it appropriate to suggest that the relevant inference _S_R^E^_A_ → _S_R^E^_C_ can be thought of as one of the functions in a recurrent neural network behaving like a *long short-term memory* (see Elman, 1990; Hochreiter and Schmidhuber, 1997). The schematic representation in Figure 3 shows the stripped-down form of a neural network of this kind that maintains the same state when passing from one step to another in backpropagation (the activation links from the input layer to the hidden layer(s) are not shown).

Figure 3 shows how _S_R^E^_A_ → _S_R^E^_C_ can be easily modeled. Here, *i_1_…i_n_* and *o_1_…o_n_* are the input and output vectors, respectively. This network at time point *k* takes *i_k_* and *o_k-1_* for its computation, when *k* can be any value within *1…n*. F in each box represents a function that can also double as a network. The inputs to this network can be thought of the sequence of terms, that is, A and C, and the outputs would be 1 or 0 indicating whether A = C or not. The output values may actually be probabilistic. Then the formulations in (18) specify how _S_R^E^_A_ → _S_R^E^_C_ can be modeled.

18. Forget(k) = Ʃ(W^F^.*o*_k-1_*i*_k_ + b^F^)

Input Valuation(k) = Ʃ(W^I^.*o*_k-1_*i*_k_ + b^I^).

Inference State(k) = Forget(k) ○ Inference State(k-1) + Input Valuation(k) ○ C^K^.

In (18) *o*_k-1_*i*_k_ is the concatenation of vectors and Ʃ as a function implements the inference _S_R^E^_A_ → _S_R^E^_C_ from the matrix of connection weights *W* between layers of the network multiplied by the value of the previous activations represented by *o*_k-1_*i*_k_ with an added bias *b*. Each *W* or *b* is indexed with the value of the processing at time point *k*. Forget(k), Input Valuation(k), and Inference State(k) are all vectors. Forget(k) determines the strength of activations in some dimension of a previous Inference State at *k-1*, and Input Valuation(k) determines the strength of input valuation when A and C are presented. Finally, Inference State(k) determines which values are to be erased in view of Forget(k) (the *element-wise multiplication* of Forget(k) and a previous Inference State at *k-*1) and which ones are to be encoded next in view of Input Valuation(k) and a fresh valuation (C^K^) vector for the Inference State at *k*. Here, the strength of _S_R^E^_A_ is fixed by Forget(k), and Inference State(k) determines the erasure of incompatible dimensions (qualia properties) with the accommodation of the valuation of _S_R^E^_C_ in B and then the consequent establishment of _S_R^E^_A_ = _S_R^E^_C_ with the fresh valuation.

In all, this discussion is meant to suggest that the coordination principles between perception and cognition in cognitive systems must include, among all other things, a strategy of creating *proxy labels* for perceptual objects such that these proxy labels can help focus on the most significant perceptual details in virtue of being marked with emotionally salient attributes/values. The emotionally salient attributes/values will constitute the task-relevant perceptual detail for the system that runs inferences of some sort. The relatively small amount of perceptual detail needed for, say, a task of picking out a red ball from a trash can is the emotionally salient valuation (for example, *solid-round-object* • *favorite-color* ⨂ *_Telic_ play-in-garden*) of a proxy representation, say, “the garden favorite” for the red ball. Likewise, an old chair in a room can be endowed with emotional attributes/values that help attend to its most relevant perceptual detail. If its proxy label is, let us suppose, “the comfort of the corner room” having the valuation *physical-object-with-four-legs* • *comfort-of-corner-room* ⨂ *_Telic_ sit-on* ⨂ *_Agentive_ make*, this can help focus solely on the emotionally salient perceptual detail of the object in question. In this way, the emotional/affective valuation attributes help identify objects of perception with the help of proxy labels in just the same way the proxy label “the guest of honor” in the event mentioned in example (6) helps identify “Mr X.” Since the perceptual world is full of complexity due to the clutter of a lot of irrelevant detail, linguistically encoded emotive representations of everyday objects, events, and things in terms of extensionally equivalent terms/labels or representations help tame this complexity, for inferences involved in establishing the identity imbue objects with *cognitive salience via* emotional valuation. Thus, perception becomes infused with cognitive representations *via* emotions. The “functional capabilities” of autonomous systems (see Langley et al., 2009) must then be integrated in a manner that ensures the affective valuation of *all* perceived objects whose representations are created and manipulated as the systems learn. This will come with the consequence that all of what the systems learn from the perceptual world becomes a matter of learning emotional/affective attributes/values of objects of perception.

## Concluding Remarks

This paper has attempted to show that linguistic representations of emotive contents can offer a unifying conceptual perspective on the problem of perception-cognition integration. It turns out that linguistic representations of emotions allowing for extensional equivalence are the ones that readily help tackle the problem of excessive computational burden of perceptual complexity the perceptual world affords. This paper has shown some directions toward this goal with the hope that further research along this line may be conducted.

## Author Contributions

The author confirms being the sole contributor of this work and has approved it for publication.

## Funding

The author wishes to acknowledge the funding received from the Cumulative Professional Development Allowance of the Indian Institute of Technology Hyderabad.

## Conflict of Interest

The author declares that the research was conducted in the absence of any commercial or financial relationships that could be construed as a potential conflict of interest.

## Publisher’s Note

All claims expressed in this article are solely those of the authors and do not necessarily represent those of their affiliated organizations, or those of the publisher, the editors and the reviewers. Any product that may be evaluated in this article, or claim that may be made by its manufacturer, is not guaranteed or endorsed by the publisher.

## Footnotes

^1^Cognitive systems are those (including humans and autonomous/computational systems) that perform cognitive tasks, such as communicating, understanding, learning, adapting, planning, deciding, problem-solving, analyzing, and judging, through integration of action and perception.

^2^It is not necessary that emotive/affective representations have to be always valenced or else they cannot count as emotive affective states. Some emotive/affective representations can be neutral in the sense that the relevant states are akin to *feeling nothing in particular*. Such neutral states have been experimentally observed to engage the working memory just like any other emotive/affective representations and also guide cognition and behavior (Gasper et al., 2019).

^3^Intentional contents are *qualitative* properties of intentionality which is characterized by the directedness of mental states toward things, events etc. (Searle, 1983)—for instance, thinking of a concert is *directed toward* the concert and the content of this thinking is the intentional content. In this sense, the experiences toward which emotions are directed are intentional contents, since emotion in the form of awareness involving global states of cooperative activity in the forebrain is essential for intentional actions (see Freeman, 2000).
